# Defining the process to literature searching in systematic reviews: a literature review of guidance and supporting studies

**DOI:** 10.1186/s12874-018-0545-3

**Published:** 2018-08-14

**Authors:** Chris Cooper, Andrew Booth, Jo Varley-Campbell, Nicky Britten, Ruth Garside

**Affiliations:** 1 Institute of Health Research, University of Exeter Medical School, Exeter, UK; 2 HEDS, School of Health and Related Research (ScHARR), University of Sheffield, Sheffield, UK; 3 Institute of Health Research, University of Exeter Medical School, Exeter, UK; 4 European Centre for Environment and Human Health, University of Exeter Medical School, Truro, UK

## Abstract

**Background:**

Systematic literature searching is recognised as a critical component of the systematic review process. It involves a systematic search for studies and aims for a transparent report of study identification, leaving readers clear about what was done to identify studies, and how the findings of the review are situated in the relevant evidence.

Information specialists and review teams appear to work from a shared and tacit model of the literature search process. How this tacit model has developed and evolved is unclear, and it has not been explicitly examined before.

The purpose of this review is to determine if a shared model of the literature searching process can be detected across systematic review guidance documents and, if so, how this process is reported in the guidance and supported by published studies.

**Method:**

A literature review.

Two types of literature were reviewed: guidance and published studies. Nine guidance documents were identified, including: The Cochrane and Campbell Handbooks. Published studies were identified through ‘pearl growing’, citation chasing, a search of PubMed using the systematic review methods filter, and the authors’ topic knowledge.

The relevant sections within each guidance document were then read and re-read, with the aim of determining key methodological stages. Methodological stages were identified and defined. This data was reviewed to identify agreements and areas of unique guidance between guidance documents. Consensus across multiple guidance documents was used to inform selection of ‘key stages’ in the process of literature searching.

**Results:**

Eight key stages were determined relating specifically to literature searching in systematic reviews. They were: who should literature search, aims and purpose of literature searching, preparation, the search strategy, searching databases, supplementary searching, managing references and reporting the search process.

**Conclusions:**

Eight key stages to the process of literature searching in systematic reviews were identified. These key stages are consistently reported in the nine guidance documents, suggesting consensus on the key stages of literature searching, and therefore the process of literature searching as a whole, in systematic reviews. Further research to determine the suitability of using the same process of literature searching for all types of systematic review is indicated.

**Electronic supplementary material:**

The online version of this article (10.1186/s12874-018-0545-3) contains supplementary material, which is available to authorized users.

## Background

Systematic literature searching is recognised as a critical component of the systematic review process. It involves a systematic search for studies and aims for a transparent report of study identification, leaving review stakeholders clear about what was done to identify studies, and how the findings of the review are situated in the relevant evidence.

Information specialists and review teams appear to work from a shared and tacit model of the literature search process. How this tacit model has developed and evolved is unclear, and it has not been explicitly examined before. This is in contrast to the information science literature, which has developed information processing models as an explicit basis for dialogue and empirical testing. Without an explicit model, research in the process of systematic literature searching will remain immature and potentially uneven, and the development of shared information models will be assumed but never articulated.

One way of developing such a conceptual model is by formally examining the implicit “programme theory” as embodied in key methodological texts. The aim of this review is therefore to determine if a shared model of the literature searching process in systematic reviews can be detected across guidance documents and, if so, how this process is reported and supported.

## Methods

### Identifying guidance

Key texts (henceforth referred to as “guidance”) were identified based upon their accessibility to, and prominence within, United Kingdom systematic reviewing practice. The United Kingdom occupies a prominent position in the science of health information retrieval, as quantified by such objective measures as the authorship of papers, the number of Cochrane groups based in the UK, membership and leadership of groups such as the Cochrane Information Retrieval Methods Group, the HTA-I Information Specialists’ Group and historic association with such centres as the UK Cochrane Centre, the NHS Centre for Reviews and Dissemination, the Centre for Evidence Based Medicine and the National Institute for Clinical Excellence (NICE). Coupled with the linguistic dominance of English within medical and health science and the science of systematic reviews more generally, this offers a justification for a purposive sample that favours UK, European and Australian guidance documents.

Nine guidance documents were identified. These documents provide guidance for different types of reviews, namely: reviews of interventions, reviews of health technologies, reviews of qualitative research studies, reviews of social science topics, and reviews to inform guidance.

Whilst these guidance documents occasionally offer additional guidance on other types of systematic reviews, we have focused on the core and stated aims of these documents as they relate to literature searching. Table 1 sets out: the guidance document, the version audited, their core stated focus, and a bibliographical pointer to the main guidance relating to literature searching.

**Table 1:** Guidance documents audited for this literature review

Guidance documents	Version: Year	Core focus	Where the guidance is reported
Systematic Reviews: CRD’s guidance for undertaking reviews in health care [6].	2009	Systematic reviews of health care interventions	1.3 Pages 16–22
The Cochrane Handbook [9].	Version 5.1: June 2017	Systematic reviews of interventions	Chapter 6: Searching for studies
Collaboration for environmental evidence: Guidelines for systematic reviews in environmental management [10].	Version 4.2 March 2013	Systematic reviews of environmental evidence	Section “Other handbooks exist” (pages 36–41)
Joanna Briggs Institute Reviewers’ Manual [11].	2014 edition	Systematic reviews of qualitative studies	Chapter 7 Information Retrieval (pages 28–31)
Institute for Quality and Efficiency in Health Care (IQWiG): IQWiG [3].	2014	Systematic reviews of health care interventions	Chapter 7: Information retrieval
Systematic Reviews in the Social Sciences: A Practical Guide [2].	2006	Systematic reviews of social science topics	Chapter 4. How to find the studies: the literature search (pages 81–124)
Process of information retrieval for systematic reviews and health technology assessments on clinical effectiveness. Eunethta [7].	Version 1.1 December 2016.	Systematic reviews of health care interventions	Standalone guideline on literature searching
The Campbell Handbook: Searching for studies: a guide to information retrieval for Campbell systematic reviews [8].	Version 1.1. February 2017.	Systematic reviews of interventions in social science topics	Standalone guideline on literature searching
Developing NICE guidelines: the manual [4].	2014	Systematic reviews to inform health care guidelines	Chapter 5. Identifying the evidence: literature searching and evidence submission.

Once a list of key guidance documents was determined, it was checked by six senior information professionals based in the UK for relevance to current literature searching in systematic reviews.

### Identifying supporting studies

**Fig. 1:**
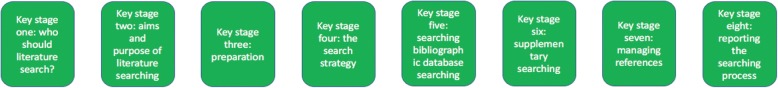
The key stages of literature search guidance as identified from nine key texts

In addition to identifying guidance, the authors sought to populate an evidence base of supporting studies (henceforth referred to as “studies”) that contribute to existing search practice. Studies were first identified by the authors from their knowledge on this topic area and, subsequently, through systematic citation chasing key studies (‘pearls’ [1]) located within each key stage of the search process. These studies are identified in Additional file 1: Appendix Table 1. Citation chasing was conducted by analysing the bibliography of references for each study (backwards citation chasing) and through Google Scholar (forward citation chasing). A search of PubMed using the systematic review methods filter was undertaken in August 2017 (see Additional file 1). The search terms used were: (literature search*[Title/Abstract]) AND sysrev_methods[sb] and 586 results were returned. These results were sifted for relevance to the key stages in Fig. 1 by CC.

### Extracting the data

To reveal the implicit process of literature searching within each guidance document, the relevant sections (chapters) on literature searching were read and re-read, with the aim of determining key methodological stages. We defined a key methodological stage as a distinct step in the overall process for which specific guidance is reported, and action is taken, that collectively would result in a completed literature search.

The chapter or section sub-heading for each methodological stage was extracted into a table using the exact language as reported in each guidance document. The lead author (CC) then read and re-read these data, and the paragraphs of the document to which the headings referred, summarising section details. This table was then reviewed, using comparison and contrast to identify agreements and areas of unique guidance. Consensus across multiple guidelines was used to inform selection of ‘key stages’ in the process of literature searching.

Having determined the key stages to literature searching, we then read and re-read the sections relating to literature searching again, extracting specific detail relating to the methodological process of literature searching within each key stage. Again, the guidance was then read and re-read, first on a document-by-document-basis and, secondly, across all the documents above, to identify both commonalities and areas of unique guidance.

## Results and discussion

### Our findings

We were able to identify consensus across the guidance on literature searching for systematic reviews suggesting a shared implicit model within the information retrieval community. Whilst the structure of the guidance varies between documents, the same key stages are reported, even where the core focus of each document is different. We were able to identify specific areas of unique guidance, where a document reported guidance not summarised in other documents, together with areas of consensus across guidance.

### Unique guidance

Only one document provided guidance on the topic of when to stop searching [2]. This guidance from 2005 anticipates a topic of increasing importance with the current interest in time-limited (i.e. “rapid”) reviews. Quality assurance (or peer review) of literature searches was only covered in two guidance documents [3, 4]. This topic has emerged as increasingly important as indicated by the development of the PRESS instrument [5]. Text mining was discussed in four guidance documents [4, 6–8] where the automation of some manual review work may offer efficiencies in literature searching [8].

### Agreement between guidance: Defining the key stages of literature searching

Where there was agreement on the process, we determined that this constituted a key stage in the process of literature searching to inform systematic reviews.

From the guidance, we determined eight key stages that relate specifically to literature searching in systematic reviews. These are summarised at Fig. 1. The data extraction table to inform Fig. 1 is reported in Table 2. Table 2 reports the areas of common agreement and it demonstrates that the language used to describe key stages and processes varies significantly between guidance documents.

**Table 2:** The order of literature search methods as presented in the guidance documents

Step	The CRD Handbook	The Cochrane Handbook	Collaboration for environmental evidence	Joanna Briggs Institute reviewers manual	IQWiG Methods Resources	Systematic reviews in the social sciences: a practical guide	Eunethta	Campbell Handbook	Developing NICE guidelines: the manual
1	Searching electronic databases	Searching bibliographic databases	Searching online literature databases and catalogues	Databases (development of search strategies, phase one)	Bibliographic databases (1.search for primary literature. 2. search for SRs)	Databases	Bibliographic databases	Bibliographic databases (1. subject databases. 2. general databases)	No list of search methods but guidance distinguishes between database searching (first) and supplementary searching (second)
2	Scanning references lists of relevant studies	Handsearching	Searching websites of organisations and professional networks	Database searching (phase two)	Search in trial registries	Grey literature	Study registries	Conference proceedings and meeting abstracts	
3	Handsearching of key journals	Conference abstracts or proceedings	Searching the world-wide web	Review reference lists	Clinical practice guideline databases and providers	identifying on-going research	Searching for unpublished company documents	Existing review and publication reference lists	
4	Searching trials registers	Other reviews	Searching bibliographies of key articles/ reviews	Handsearching	Requests to manufacturers	Theses	Regulatory documents	Web searching	
5	Contacting experts and manufactures	Web-searching	Contacting key individuals who work in the area		Other data sources	Conference proceedings	Queries to authors	Unpublished studies	
6	Searching relevant internet resources	Unpublished and on-going studies (inc. author contact)	Citation searches for key papers/ included papers			Citation searching	Further search techniques	On-going studies	
7	Citation searching					Searching the web		Institutional repositories	
8	Using a project website to canvas for studies					contact with experts		handsearching	
9						Trials registers			

For each key stage, we set out the specific guidance, followed by discussion on how this guidance is situated within the wider literature.

### Key stage one: Deciding who should undertake the literature search

#### The guidance

Eight documents provided guidance on who should undertake literature searching in systematic reviews [2, 4, 6–11]. The guidance affirms that people with relevant expertise of literature searching should ‘ideally’ be included within the review team [6]. Information specialists (or information scientists), librarians or trial search co-ordinators (TSCs) are indicated as appropriate researchers in six guidance documents [2, 7–11].

#### How the guidance corresponds to the published studies

The guidance is consistent with studies that call for the involvement of information specialists and librarians in systematic reviews [12–26] and which demonstrate how their training as ‘expert searchers’ and ‘analysers and organisers of data’ can be put to good use [13] in a variety of roles [12, 16, 20, 21, 24–26]. These arguments make sense in the context of the aims and purposes of literature searching in systematic reviews, explored below. The need for ‘thorough’ and ‘replicable’ literature searches was fundamental to the guidance and recurs in key stage two. Studies have found poor reporting, and a lack of replicable literature searches, to be a weakness in systematic reviews [17, 18, 27, 28] and they argue that involvement of information specialists/ librarians would be associated with better reporting and better quality literature searching. Indeed, Meert et al. [29] demonstrated that involving a librarian as a co-author to a systematic review correlated with a higher score in the literature searching component of a systematic review [29]. As ‘new styles’ of rapid and scoping reviews emerge, where decisions on how to search are more iterative and creative, a clear role is made here too [30].

Knowing where to search for studies was noted as important in the guidance, with no agreement as to the appropriate number of databases to be searched [2, 6]. Database (and resource selection more broadly) is acknowledged as a relevant key skill of information specialists and librarians [12, 15, 16, 31].

Whilst arguments for including information specialists and librarians in the process of systematic review might be considered self-evident, Koffel and Rethlefsen [31] have questioned if the necessary involvement is actually happening [31].

### Key stage two: Determining the aim and purpose of a literature search

#### The guidance

**The aim:** Five of the nine guidance documents use adjectives such as ‘thorough’, ‘comprehensive’, ‘transparent’ and ‘reproducible’ to define the aim of literature searching [6–10]. Analogous phrases were present in a further three guidance documents, namely: ‘to identify the best available evidence’ [4] or ‘the aim of the literature search is not to retrieve everything. It is to retrieve everything of relevance’ [2] or ‘A systematic literature search aims to identify all publications relevant to the particular research question’ [3]. The Joanna Briggs Institute reviewers’ manual was the only guidance document where a clear statement on the aim of literature searching could not be identified. The purpose of literature searching was defined in three guidance documents, namely to minimise bias in the resultant review [6, 8, 10]. Accordingly, eight of nine documents clearly asserted that thorough and comprehensive literature searches are required as a potential mechanism for minimising bias.

##### How the guidance corresponds to the published studies

The need for thorough and comprehensive literature searches appears as uniform within the eight guidance documents that describe approaches to literature searching in systematic reviews of effectiveness. Reviews of effectiveness (of intervention or cost), accuracy and prognosis, require thorough and comprehensive literature searches to transparently produce a reliable estimate of intervention effect. The belief that all relevant studies have been ‘comprehensively’ identified, and that this process has been ‘transparently’ reported, increases confidence in the estimate of effect and the conclusions that can be drawn [32]. The supporting literature exploring the need for comprehensive literature searches focuses almost exclusively on reviews of intervention effectiveness and meta-analysis. Different ‘styles’ of review may have different standards however; the alternative, offered by purposive sampling, has been suggested in the specific context of qualitative evidence syntheses [33].

#### What is a comprehensive literature search?

Whilst the guidance calls for thorough and comprehensive literature searches, it lacks clarity on what constitutes a thorough and comprehensive literature search, beyond the implication that all of the literature search methods in Table 2 should be used to identify studies. Egger et al. [34], in an empirical study evaluating the importance of comprehensive literature searches for trials in systematic reviews, defined a comprehensive search for trials as:

- a search not restricted to English language;
- where Cochrane CENTRAL or at least two other electronic databases had been searched (such as MEDLINE or EMBASE); and
- at least one of the following search methods has been used to identify unpublished trials: searches for (I) conference abstracts, (ii) theses, (iii) trials registers; and (iv) contacts with experts in the field [34].

Tricco et al. (2008) used a similar threshold of bibliographic database searching AND a supplementary search method in a review when examining the risk of bias in systematic reviews. Their criteria were: one database (limited using the Cochrane Highly Sensitive Search Strategy (HSSS)) and handsearching [35].

Together with the guidance, this would suggest that comprehensive literature searching requires the use of BOTH bibliographic database searching AND supplementary search methods.

Comprehensiveness in literature searching, in the sense of how much searching should be undertaken, remains unclear. Egger et al. recommend that ‘investigators should consider the type of literature search and degree of comprehension that is appropriate for the review in question, taking into account budget and time constraints’ [34]. This view tallies with the Cochrane Handbook, which stipulates clearly, that study identification should be undertaken ‘within resource limits’ [9]. This would suggest that the limitations to comprehension are recognised but it raises questions on how this is decided and reported [36].

##### What is the point of comprehensive literature searching?

The purpose of thorough and comprehensive literature searches is to avoid missing key studies and to minimize bias [6, 8, 10, 34, 37–39] since a systematic review based only on published (or easily accessible) studies may have an exaggerated effect size [35]. Felson (1992) sets out potential biases that could affect the estimate of effect in a meta-analysis [40] and Tricco et al. summarize the evidence concerning bias and confounding in systematic reviews [35]. Egger et al. point to non-publication of studies, publication bias, language bias and MEDLINE bias, as key biases [34, 35, 40–46]. Comprehensive searches are not the sole factor to mitigate these biases but their contribution is thought to be significant [2, 32, 34]. Fehrmann (2011) suggests that ‘the search process being described in detail’ and that, where standard comprehensive search techniques have been applied, increases confidence in the search results [32].

##### Does comprehensive literature searching work?

Egger et al., and other study authors, have demonstrated a change in the estimate of intervention effectiveness where relevant studies were excluded from meta-analysis [34, 47]. This would suggest that missing studies in literature searching alters the reliability of effectiveness estimates. This is an argument for comprehensive literature searching. Conversely, Egger et al. found that ‘comprehensive’ searches still missed studies and that comprehensive searches could, in fact, introduce bias into a review rather than preventing it, through the identification of low quality studies then being included in the meta-analysis [34]. Studies query if identifying and including low quality or grey literature studies changes the estimate of effect [43, 48] and question if time is better invested updating systematic reviews rather than searching for unpublished studies [49], or mapping studies for review as opposed to aiming for high sensitivity in literature searching [50].

##### Aim and purpose beyond reviews of effectiveness

The need for comprehensive literature searches is less certain in reviews of qualitative studies, and for reviews where a comprehensive identification of studies is difficult to achieve (for example, in Public health) [33, 51–55]. Literature searching for qualitative studies, and in public health topics, typically generates a greater number of studies to sift than in reviews of effectiveness [39] and demonstrating the ‘value’ of studies identified or missed is harder [56], since the study data do not typically support meta-analysis. Nussbaumer-Streit et al. (2016) have registered a review protocol to assess whether abbreviated literature searches (as opposed to comprehensive literature searches) has an impact on conclusions across multiple bodies of evidence, not only on effect estimates [57] which may develop this understanding. It may be that decision makers and users of systematic reviews are willing to trade the certainty from a comprehensive literature search and systematic review in exchange for different approaches to evidence synthesis [58], and that comprehensive literature searches are not necessarily a marker of literature search quality, as previously thought [36]. Different approaches to literature searching [37, 38, 59–62] and developing the concept of when to stop searching are important areas for further study [36, 59].

The study by Nussbaumer-Streit et al. has been published since the submission of this literature review [63]. Nussbaumer-Streit et al. (2018) conclude that abbreviated literature searches are viable options for rapid evidence syntheses, if decision-makers are willing to trade the certainty from a comprehensive literature search and systematic review, but that decision-making which demands detailed scrutiny should still be based on comprehensive literature searches [63].

### Key stage three: Preparing for the literature search

#### The guidance

Six documents provided guidance on preparing for a literature search [2, 3, 6, 7, 9, 10]. The Cochrane Handbook clearly stated that Cochrane authors (i.e. researchers) should seek advice from a trial search co-ordinator (i.e. a person with specific skills in literature searching) ‘before’ starting a literature search [9].

Two key tasks were perceptible in preparing for a literature searching [2, 6, 7, 10, 11]. First, to determine if there are any existing or on-going reviews, or if a new review is justified [6, 11]; and, secondly, to develop an initial literature search strategy to estimate the volume of relevant literature (and quality of a small sample of relevant studies [10]) and indicate the resources required for literature searching and the review of the studies that follows [7, 10].

Three documents summarised guidance on where to search to determine if a new review was justified [2, 6, 11]. These focused on searching databases of systematic reviews (The Cochrane Database of Systematic Reviews (CDSR) and the Database of Abstracts of Reviews of Effects (DARE)), institutional registries (including PROSPERO), and MEDLINE [6, 11]. It is worth noting, however, that as of 2015, DARE (and NHS EEDs) are no longer being updated and so the relevance of this (these) resource(s) will diminish over-time [64]. One guidance document, ‘Systematic reviews in the Social Sciences’, noted, however, that databases are not the only source of information and unpublished reports, conference proceeding and grey literature may also be required, depending on the nature of the review question [2].

Two documents reported clearly that this preparation (or ‘scoping’) exercise should be undertaken before the actual search strategy is developed [7, 10]).

#### How the guidance corresponds to the published studies

The guidance offers the best available source on preparing the literature search with the published studies not typically reporting how their scoping informed the development of their search strategies nor how their search approaches were developed. Text mining has been proposed as a technique to develop search strategies in the scoping stages of a review although this work is still exploratory [65]. ‘Clustering documents’ and word frequency analysis have also been tested to identify search terms and studies for review [66, 67]. Preparing for literature searches and scoping constitutes an area for future research.

### Key stage four: Designing the search strategy

#### The guidance

The Population, Intervention, Comparator, Outcome (PICO) structure was the commonly reported structure promoted to design a literature search strategy. Five documents suggested that the eligibility criteria or review question will determine which concepts of PICO will be populated to develop the search strategy [1, 4, 7–9]. The NICE handbook promoted multiple structures, namely PICO, SPICE (Setting, Perspective, Intervention, Comparison, Evaluation) and multi-stranded approaches [4].

With the exclusion of The Joanna Briggs Institute reviewers’ manual, the guidance offered detail on selecting key search terms, synonyms, Boolean language, selecting database indexing terms and combining search terms. The CEE handbook suggested that ‘search terms may be compiled with the help of the commissioning organisation and stakeholders’ [10].

The use of limits, such as language or date limits, were discussed in all documents [2–4, 6–11].

### How the guidance corresponds to the published studies

#### Search strategy structure

The guidance typically relates to reviews of intervention effectiveness so PICO – with its focus on intervention and comparator - is the dominant model used to structure literature search strategies [68]. PICOs – where the S denotes study design - is also commonly used in effectiveness reviews [6, 68]. As the NICE handbook notes, alternative models to structure literature search strategies have been developed and tested. Booth provides an overview on formulating questions for evidence based practice [69] and has developed a number of alternatives to the PICO structure, namely: BeHEMoTh (Behaviour of interest; Health context; Exclusions; Models or Theories) for use when systematically identifying theory [55]; SPICE (Setting, Perspective, Intervention, Comparison, Evaluation) for identification of social science and evaluation studies [69] and, working with Cooke and colleagues, SPIDER (Sample, Phenomenon of Interest, Design, Evaluation, Research type) [70]. SPIDER has been compared to PICO and PICOs in a study by Methley et al. [68].

The NICE handbook also suggests the use of multi-stranded approaches to developing literature search strategies [4]. Glanville developed this idea in a study by Whitting et al. [71] and a worked example of this approach is included in the development of a search filter by Cooper et al. [72].

#### Writing search strategies: Conceptual and objective approaches

Hausner et al. [73] provide guidance on writing literature search strategies, delineating between conceptually and objectively derived approaches. The conceptual approach, advocated by and explained in the guidance documents, relies on the expertise of the literature searcher to identify key search terms and then develop key terms to include synonyms and controlled syntax. Hausner and colleagues set out the objective approach [73] and describe what may be done to validate it [74].

#### The use of limits

The guidance documents offer direction on the use of limits within a literature search. Limits can be used to focus literature searching to specific study designs or by other markers (such as by date) which limits the number of studies returned by a literature search. The use of limits should be described and the implications explored [34] since limiting literature searching can introduce bias (explored above). Craven et al. have suggested the use of a supporting narrative to explain decisions made in the process of developing literature searches and this advice would usefully capture decisions on the use of search limits [75].

### Key stage five: Determining the process of literature searching and deciding where to search (bibliographic database searching)

#### The guidance

Table 2 summarises the process of literature searching as reported in each guidance document. Searching bibliographic databases was consistently reported as the ‘first step’ to literature searching in all nine guidance documents.

Three documents reported specific guidance on where to search, in each case specific to the type of review their guidance informed, and as a minimum requirement [4, 9, 11]. Seven of the key guidance documents suggest that the selection of bibliographic databases depends on the topic of review [2–4, 6–8, 10], with two documents noting the absence of an agreed standard on what constitutes an acceptable number of databases searched [2, 6].

#### How the guidance corresponds to the published studies

The guidance documents summarise ‘how to’ search bibliographic databases in detail and this guidance is further contextualised above in terms of developing the search strategy. The documents provide guidance of selecting bibliographic databases, in some cases stating acceptable minima (i.e. The Cochrane Handbook states Cochrane CENTRAL, MEDLINE and EMBASE), and in other cases simply listing bibliographic database available to search. Studies have explored the value in searching specific bibliographic databases, with Wright et al. (2015) noting the contribution of CINAHL in identifying qualitative studies [76], Beckles et al. (2013) questioning the contribution of CINAHL to identifying clinical studies for guideline development [77], and Cooper et al. (2015) exploring the role of UK-focused bibliographic databases to identify UK-relevant studies [78]. The host of the database (e.g. OVID or ProQuest) has been shown to alter the search returns offered. Younger and Boddy [79] report differing search returns from the same database (AMED) but where the ‘host’ was different [79].

The average number of bibliographic database searched in systematic reviews has risen in the period 1994–2014 (from 1 to 4) [80] but there remains (as attested to by the guidance) no consensus on what constitutes an acceptable number of databases searched [48]. This is perhaps because thinking about *the number* of databases searched is the wrong question, researchers should be focused on which databases were searched and why, and which databases were not searched and why. The discussion should re-orientate to the differential value of sources but researchers need to think about how to report this in studies to allow findings to be generalised. Bethel (2017) has proposed ‘search summaries’, completed by the literature searcher, to record where included studies were identified, whether from database (and which databases specifically) or supplementary search methods [81]. Search summaries document both yield and accuracy of searches, which could prospectively inform resource use and decisions to search or not to search specific databases in topic areas. The prospective use of such data presupposes, however, that past searches are a potential predictor of future search performance (i.e. that each topic is to be considered representative and not unique). In offering a body of practice, this data would be of greater practicable use than current studies which are considered as little more than individual case studies [82–90].

When to database search is another question posed in the literature. Beyer et al. [91] report that databases can be prioritised for literature searching which, whilst not addressing the question of which databases to search, may at least bring clarity as to which databases to search first [91]. Paradoxically, this links to studies that suggest PubMed should be searched in addition to MEDLINE (OVID interface) since this improves the currency of systematic reviews [92, 93]. Cooper et al. (2017) have tested the idea of database searching not as a primary search method (as suggested in the guidance) but as a supplementary search method in order to manage the volume of studies identified for an environmental effectiveness systematic review. Their case study compared the effectiveness of database searching versus a protocol using supplementary search methods and found that the latter identified more relevant studies for review than searching bibliographic databases [94].

### Key stage six: Determining the process of literature searching and deciding where to search (supplementary search methods)

#### The guidance

Table 2 also summaries the process of literature searching which follows bibliographic database searching. As Table 2 sets out, guidance that supplementary literature search methods should be used in systematic reviews recurs across documents, but the order in which these methods are used, and the extent to which they are used, varies. We noted inconsistency in the labelling of supplementary search methods between guidance documents.

#### How the guidance corresponds to the published studies

Rather than focus on the guidance on how to use the methods (which has been summarised in a recent review [95]), we focus on the aim or purpose of supplementary search methods.

The Cochrane Handbook reported that ‘efforts’ to identify unpublished studies should be made [9]. Four guidance documents [2, 3, 6, 9] acknowledged that searching beyond bibliographic databases was necessary since ‘databases are not the only source of literature’ [2]. Only one document reported any guidance on determining when to use supplementary methods. The IQWiG handbook reported that the use of handsearching (in their example) could be determined on a ‘case-by-case basis’ which implies that the use of these methods is optional rather than mandatory. This is in contrast to the guidance (above) on bibliographic database searching.

The issue for supplementary search methods is similar in many ways to the issue of searching bibliographic databases: demonstrating value. The purpose and contribution of supplementary search methods in systematic reviews is increasingly acknowledged [37, 61, 62, 96–101] but understanding the value of the search methods to identify studies and data is unclear. In a recently published review, Cooper et al. (2017) reviewed the literature on supplementary search methods looking to determine the advantages, disadvantages and resource implications of using supplementary search methods [95]. This review also summarises the key guidance and empirical studies and seeks to address the question on when to use these search methods and when not to [95]. The guidance is limited in this regard and, as Table 2 demonstrates, offers conflicting advice on the order of searching, and the extent to which these search methods should be used in systematic reviews.

### Key stage seven: Managing the references

#### The guidance

Five of the documents provided guidance on managing references, for example downloading, de-duplicating and managing the output of literature searches [2, 4, 6, 8, 10]. This guidance typically itemised available bibliographic management tools rather than offering guidance on how to use them specifically [2, 4, 6, 8]. The CEE handbook provided guidance on importing data where no direct export option is available (e.g. web-searching) [10].

#### How the guidance corresponds to the published studies

The literature on using bibliographic management tools is not large relative to the number of ‘how to’ videos on platforms such as YouTube (see for example [102]). These YouTube videos confirm the overall lack of ‘how to’ guidance identified in this study and offer useful instruction on managing references. Bramer et al. set out methods for de-duplicating data and reviewing references in Endnote [103, 104] and Gall tests the direct search function within Endnote to access databases such as PubMed, finding a number of limitations [105]. Coar et al. and Ahmed et al. consider the role of the free-source tool, Zotero [106, 107]. Managing references is a key administrative function in the process of review particularly for documenting searches in PRISMA guidance.

### Key stage eight: Documenting the search

#### The guidance

The Cochrane Handbook was the only guidance document to recommend a specific reporting guideline: Preferred Reporting Items for Systematic Reviews and Meta-Analyses (PRISMA) [9]. Six documents provided guidance on reporting the process of literature searching with specific criteria to report [3, 4, 6, 8–10]. There was consensus on reporting: the databases searched (and the host searched by), the search strategies used, and any use of limits (e.g. date, language, search filters (The CRD handbook called for these limits to be justified [6])). Three guidance documents reported that the number of studies identified should be recorded [3, 6, 10]. The number of duplicates identified [10], the screening decisions [3], a comprehensive list of grey literature sources searched (and full detail for other supplementary search methods) [8], and an annotation of search terms tested but not used [4] were identified as unique items in four documents.

The Cochrane Handbook was the only guidance document to note that the full search strategies for each database should be included in the Additional file 1 of the review [9].

#### How the guidance corresponds to the published studies

All guidance documents should ultimately deliver completed systematic reviews that fulfil the requirements of the PRISMA reporting guidelines [108]. The guidance broadly requires the reporting of data that corresponds with the requirements of the PRISMA statement although documents typically ask for diverse and additional items [108]. In 2008, Sampson et al. observed a lack of consensus on reporting search methods in systematic reviews [109] and this remains the case as of 2017, as evidenced in the guidance documents, and in spite of the publication of the PRISMA guidelines in 2009 [110]. It is unclear why the collective guidance does not more explicitly endorse adherence to the PRISMA guidance.

Reporting of literature searching is a key area in systematic reviews since it sets out clearly what was done and how the conclusions of the review can be believed [52, 109]. Despite strong endorsement in the guidance documents, specifically supported in PRISMA guidance, and other related reporting standards too (such as ENTREQ for qualitative evidence synthesis, STROBE for reviews of observational studies), authors still highlight the prevalence of poor standards of literature search reporting [31, 110–119]. To explore issues experienced by authors in reporting literature searches, and look at uptake of PRISMA, Radar et al. [120] surveyed over 260 review authors to determine common problems and their work summaries the practical aspects of reporting literature searching [120]. Atkinson et al. [121] have also analysed reporting standards for literature searching, summarising recommendations and gaps for reporting search strategies [121].

One area that is less well covered by the guidance, but nevertheless appears in this literature, is the quality appraisal or peer review of literature search strategies. The PRESS checklist is the most prominent and it aims to develop evidence-based guidelines to peer review of electronic search strategies [5, 122, 123]. A corresponding guideline for documentation of supplementary search methods does not yet exist although this idea is currently being explored.

How the reporting of the literature searching process corresponds to critical appraisal tools is an area for further research. In the survey undertaken by Radar et al. (2014), 86% of survey respondents (153/178) identified a need for further guidance on what aspects of the literature search process to report [120]. The PRISMA statement offers a brief summary of what to report but little practical guidance on how to report it [108]. Critical appraisal tools for systematic reviews, such as AMSTAR 2 (Shea et al. [124]) and ROBIS (Whiting et al. [125]), can usefully be read alongside PRISMA guidance, since they offer greater detail on how the reporting of the literature search will be appraised and, therefore, they offer a proxy on what to report [124, 125]. Further research in the form of a study which undertakes a comparison between PRISMA and quality appraisal checklists for systematic reviews would seem to begin addressing the call, identified by Radar et al., for further guidance on what to report [120].

## Limitations

### Other handbooks exist

A potential limitation of this literature review is the focus on guidance produced in Europe (the UK specifically) and Australia. We justify the decision for our selection of the nine guidance documents reviewed in this literature review in section “Identifying guidance”. In brief, these nine guidance documents were selected as the most relevant health care guidance that inform UK systematic reviewing practice, given that the UK occupies a prominent position in the science of health information retrieval. We acknowledge the existence of other guidance documents, such as those from North America (e.g. the Agency for Healthcare Research and Quality (AHRQ) [126], The Institute of Medicine [127] and the guidance and resources produced by the Canadian Agency for Drugs and Technologies in Health (CADTH) [128]). We comment further on this directly below.

### The handbooks are potentially linked to one another

What is not clear is the extent to which the guidance documents inter-relate or provide guidance uniquely. The Cochrane Handbook, first published in 1994, is notably a key source of reference in guidance and systematic reviews beyond Cochrane reviews. It is not clear to what extent broadening the sample of guidance handbooks to include North American handbooks, and guidance handbooks from other relevant countries too, would alter the findings of this literature review or develop further support for the process model. Since we cannot be clear, we raise this as a potential limitation of this literature review. On our initial review of a sample of North American, and other, guidance documents (before selecting the guidance documents considered in this review), however, we do not consider that the inclusion of these further handbooks would alter significantly the findings of this literature review.

### This is a literature review

A further limitation of this review was that the review of published studies is not a systematic review of the evidence for each key stage. It is possible that other relevant studies could help contribute to the exploration and development of the key stages identified in this review.

## Conclusions

This literature review would appear to demonstrate the existence of a shared model of the literature searching process in systematic reviews. We call this model ‘the conventional approach’, since it appears to be common convention in nine different guidance documents.

The findings reported above reveal eight key stages in the process of literature searching for systematic reviews. These key stages are consistently reported in the nine guidance documents which suggests consensus on the key stages of literature searching, and therefore the process of literature searching as a whole, in systematic reviews.

In Table 2, we demonstrate consensus regarding the application of literature search methods. All guidance documents distinguish between primary and supplementary search methods. Bibliographic database searching is consistently the first method of literature searching referenced in each guidance document. Whilst the guidance uniformly supports the use of supplementary search methods, there is little evidence for a consistent process with diverse guidance across documents. This may reflect differences in the core focus across each document, linked to differences in identifying effectiveness studies or qualitative studies, for instance.

Eight of the nine guidance documents reported on the aims of literature searching. The shared understanding was that literature searching should be thorough and comprehensive in its aim and that this process should be reported transparently so that that it could be reproduced. Whilst only three documents explicitly link this understanding to minimising bias, it is clear that comprehensive literature searching is implicitly linked to ‘not missing relevant studies’ which is approximately the same point.

Defining the key stages in this review helps categorise the scholarship available, and it prioritises areas for development or further study. The supporting studies on preparing for literature searching (key stage three, ‘preparation’) were, for example, comparatively few, and yet this key stage represents a decisive moment in literature searching for systematic reviews. It is where search strategy structure is determined, search terms are chosen or discarded, and the resources to be searched are selected. Information specialists, librarians and researchers, are well placed to develop these and other areas within the key stages we identify.

This review calls for further research to determine the suitability of using the conventional approach. The publication dates of the guidance documents which underpin the conventional approach may raise questions as to whether the process which they each report remains valid for current systematic literature searching. In addition, it may be useful to test whether it is desirable to use the same process model of literature searching for qualitative evidence synthesis as that for reviews of intervention effectiveness, which this literature review demonstrates is presently recommended best practice.

## Additional file

Additional file 1: Appendix tables and PubMed search strategy. Key studies used for pearl growing per key stage, working data extraction tables and the PubMed search strategy. (DOCX 30 kb)

## Abbreviations

BeHEMoTh: Behaviour of interest; Health context; Exclusions; Models or Theories
CDSR: Cochrane Database of Systematic Reviews
Cochrane CENTRAL: The Cochrane Central Register of Controlled Trials
DARE: Database of Abstracts of Reviews of Effects
ENTREQ: Enhancing transparency in reporting the synthesis of qualitative research
IQWiG: Institute for Quality and Efficiency in Healthcare
NICE: National Institute for Clinical Excellence
PICO: Population, Intervention, Comparator, Outcome
PRISMA: Preferred Reporting Items for Systematic Reviews and Meta-Analyses
SPICE: Setting, Perspective, Intervention, Comparison, Evaluation
SPIDER: Sample, Phenomenon of Interest, Design, Evaluation, Research type
STROBE: STrengthening the Reporting of OBservational studies in Epidemiology
TSC: Trial Search Co-ordinators
