# Supplementary material for: Defining the process to literature searching in systematic reviews: a literature review of guidance and supporting studies
Source: BMC Med Res Methodol. 2018 Aug 14;18:85. doi: 10.1186/s12874-018-0545-3 (PMC6092796; doi:10.1186/s12874-018-0545-3)
Supplement: Supplementary file 1 — Appendix tables and PubMed search strategy. Key studies used for pearl growing per key stage, working data extraction tables and the PubMed search strategy. (DOCX 30 kb) [file 12874_2018_545_MOESM1_ESM.docx]

# Appendix tables and PubMed search strategy

**Table one: Table of key (pearl) studies per key stage**

| Key stage 1 | Beverley CA, Booth A, Bath PA. The role of the information specialist in the systematic review process: a health information case study. Health information and libraries journal. 2003;20(2):65-74.  McGowan J, Sampson M. Systematic reviews need systematic searchers. Journal of the Medical Library Association. 2005;93(1):74-80.  Meert D, Torabi N, Costella J. Impact of librarians on reporting of the literature searching component of pediatric systematic reviews. Journal of the Medical Library Association : JMLA. 2016;104(4):267-77. |
| --- | --- |
| Key stage 2 | Egger M, Juni P, Bartlett C, Holenstein F, Sterne J. How important are comprehensive literature searches and the assessment of trial quality in systematic reviews? Empirical study. Health technology assessment (Winchester, England). 2003;7(1):1-76.  Fehrmann P, Thomas J. Comprehensive computer searches and reporting in systematic reviews. Research Synthesis Methods. 2011;2(1):15-32. |
| Key stage 3 | No studies identified |
| Key Sage 4 | Methley AM, Campbell S, Chew-Graham C, McNally R, Cheraghi-Sohi S. PICO, PICOS and SPIDER: a comparison study of specificity and sensitivity in three search tools for qualitative systematic reviews. BMC Health Services Research. 2014;14:579.  Andrew Booth. Clear and present questions: formulating questions for evidence based practice. Library Hi Tech. 2006;24(3):355-68.  Cooke A, Smith D, Booth A. Beyond PICO: the SPIDER tool for qualitative evidence synthesis. Qual Health Res. 2012;22(10):1435-43. |
| Key stage 5 | Cooper C, Booth A, Britten N, Garside R. A comparison of results of empirical studies of supplementary search techniques and recommendations in review methodology handbooks: A methodological review. BMC Systematic Reviews (In Press) |
| Key stage 6 | Booth A. Unpacking your literature search toolbox: on search styles and tactics. Health Information & Libraries Journal. 2008;25(4):313-7. |
| Key stage 7 | Bramer WM, Giustini D, de Jonge GB, Holland L, Bekhuis T. De-duplication of database search results for systematic reviews in EndNote. J Med Libr Assoc. 2016;104(3):240-3. |
| Key stage 8 | Rader T, Mann M, Stansfield C, Cooper C, Sampson M. Methods for documenting systematic review searches: a discussion of common issues. Research Synthesis Methods. 2014;5(2):98-115.  McGowan J, Sampson M, Salzwedel DM, Cogo E, Foerster V, Lefebvre C. PRESS Peer Review of Electronic Search Strategies: 2015 Guideline Statement. J Clin Epidemiol. 2016;75:40-6.  Sampson M, McGowan J, Cogo E, Grimshaw J, Moher D, Lefebvre C. An evidence-based practice guideline for the peer review of electronic search strategies. J Clin Epidemiol. 2009;62(9):944-52.  Sampson M MJ, Lefebvre C, Moher D, Ramshaw J. PRESS: Peer Review of Electronic Search Strategies. 2008. |

|  | **The CRD Handbook** | **The Cochrane Handbook** | **Collaboration for environmental evidence** | **Joanna Briggs reviewers manual** | **IQWiG Methods Resources** | **Systematic reviews in the social sciences : a practical guide** | **Eunethta^a^** | **Campbell Handbook** | **Developing NICE guidelines: the manual** |
| --- | --- | --- | --- | --- | --- | --- | --- | --- | --- |
| Purpose of guidance | Reviews of healthcare interventions* | Reviews of interventions* | Reviews of environmental evidence | Reviews of qualitative studies* | Reviews of healthcare interventions | Reviews in social topics | Reviews of health technologies | Reviews of social sciences | Developing NICE guidance |
| Purpose of literature searching | Conducting a **thorough** search to identify relevant studies is a key factor in minimizing bias in the review process. The search process should be as **transparent** as possible and documented in a way that enables it to be evaluated and reproduced. | Systematic reviews of interventions require a **thorough,** objective and **reproducible** search of a range of sources to identify as many relevant studies as possible (within resource limits). | Systematic and **comprehensive** searching for relevant studies is essential to minimise publication bias in a SR and to assess the strength of the evidence case | The search strategy aims to find both published and unpublished studies. | A systematic literature search aims to identify **all publications relevant** to the particular research question (i.e. publications that contribute to a gain in knowledge on the topic). The search for primary literature is normally orientated towards the aim of achieving high sensitivity. | The aim of the literature search is **not to retrieve everything. It is to retrieve everything of relevance**, while leaving behind the irrelevant. | Information retrieval for systematic reviews needs to be performed in a **thorough, transparent and reproducible manner.** The aim is to identify all relevant studies and study results on the question of interest (within resource limits) [3]. This requires both searches in several information sources and the use of comprehensive search strategies [3-5]. This approach is a key factor in minimizing bias in the review process [5]. | Systematic reviews of interventions require a **thorough, objective and reproducible** search of a range of sources to identify as many relevant studies as possible (within resource limits). | The aim is to **identify the best available evidence** to address a particular question without producing an unmanageable volume of results. |
| Who should undertake literature searching | Pg 4 1.1.1 The review team  ‘Ideally these should include expertise in systematic review methods, information retrieval,’ | Section 6.1 Introduction  ‘Cochrane Review Groups (CRGs) are responsible for providing review authors with references to studies that are possibly relevant to their review. The majority of CRGs employ a dedicated Trials Search Co-ordinator to provide this service’  6.1.1.1 Role of the Trials Search Co-ordinator  ‘The Trials Search Co-ordinator for each CRG is responsible for providing assistance to authors with searching for studies for inclusion in their reviews. The range of assistance varies according to the resources available to individual CRGs but may include some or all of the following: providing relevant studies from the CRG’s Specialized Register (see Section [6.3.2.4](http://handbook-5-1.cochrane.org/chapter_6/6_3_2_4_what_is_in_the_cochrane_central_register_of_controlled.htm) for more detail), designing search strategies for the main bibliographic databases, running these searches in databases available to the CRG, saving search results and sending them to authors, advising authors on how to run searches in other databases and how to download results into their reference management software’  section 6.3.1 provides guidance on how involve trials search co-ordinators | Pg 28  'enlisting an information specialist in the review team is recommended so that an efficient search strategy can be established.’ | Pg 57  'if possible, authors should always seek the advice of a research librarian in the construction of a search strategy.' | No data reported | Pg 85  It will be clear from the above description that there are particular skills involved in carrying out sensitive and specific searches, and these tend not to be part of the core training of social researchers. This is why systematic reviewers usually seek expert help from an information scientist, as these specialists are trained in searching electronic and other sources. They will know which databases are available and how to access them through the various interfaces and providers, as well as differences between them with respect to search terms and indexing. They are also skilled in locating gray literature. | Pg 14  Information specialists should form an integral part of the project team of a systematic review from the beginning of the project. | Pg 8  This guide’s fundamental premise is that information retrieval is an essential component of the systematic review process, analogous to the data collection phase of a primary research study, and requires the expertise of TSC, an information specialist (IS) or a librarian. | No Data reported |
| Preparation/ Scoping | Pg 3  Determining if there are already existing or ongoing review or if a new review is justified. Guidance on where to scope is provided. | 6.1.2  Cochrane review authors should seek advice from the Trials Search Co-ordinator of their Cochrane Review Group (CRG) *before* starting a search. | Pg 26-27  A thorough scope should entail:  The development and testing of a search strategy.  An estimate of the volume of relevant literature.  Critical appraisal of study quality and data extraction of a small subset of relevant  papers.  An estimate of resources required based on the above.  The expected output from a scoping exercise is an estimate of the quantity and quality of  evidence, and a characterisation of the likely evidence base, pertaining to the question  (see Box 3 for example). The extent of investment in review scoping is a matter of  judgement and will differ with each review. | Pg 11  In order to avoid duplication, reviewers are advised to register their review title as mentioned previously. It is also recommended that reviewers search major  electronic databases to determine that there have been no recently published systematic reviews on the same topic prior to registration of a review title. A search of the Joanna Briggs Institute  Library of Systematic Review Protocols, Joanna Briggs Institute Library of Systematic Reviews,  Cochrane Library, MEDLINE, PROSPERO and DARE databases will assist to establish whether or not a recent review report exists on the topic of interest. | Not reported | Pg 80  ‘There are many sources of information to consider, but a search of electronic databases is often the main starting point. However such databases are not the only source of literature, and sometimes they are not even the most useful.’  ‘The type of information being sought will depend on both the review question and the inclusion criteria.’  Guidance on how to broaden these ‘scoping’ searches is given, including: searching unpublished reports, conference proceedings and gray literature. The advice that searches should include book chapters is unique. | Section 2.3.2 Conducting preliminary searches  ‘At the start of a project – before the development of the actual search strategy – a preliminary search (also known as a scoping search) should be conducted. This preliminary search has various goals.‘  ‘Firstly, to help prepare the overall project [32], i.e. understanding the key questions [4], identifying existing systematic reviews [5,50,51], identifying a first set of potentially relevant primary studies [52], and estimating the resources necessary to perform the systematic review [50]. Secondly, the results of the preliminary search can be used in the development of the search strategy, for instance, by generating a list of search terms from the analysis of identified relevant articles [4,53-55] and subsequently used in the development of the search strategy.’  ‘The most effective way of conducting a preliminary search is first to search for systematic reviews.’ | Not reported | Not reported |
| The search strategy | Pg 19  PICOs  Pg 12  ‘Whenever feasible, all relevant studies should be included regardless of language. However, realistically this is not always possible due to a lack of time, resources and facilities for translation.’ | 6.4.1  ‘The eligibility criteria for studies to be included in the review will inform how the search is conducted. The eligibility criteria will specify the types of designs, types of participants, types of intervention (experimental and comparator) and, in some cases, the types of outcomes to be addressed.’ | Pg 28  ‘This may include considering synonyms, alternative spellings, and non-English language terms within the search strategy. An initial list of search terms may  be compiled with the help of the commissioning organisation and stakeholders.’  Pg 29  ‘A final step in the development of the search terms  is to test the strategy with a set of  known relevant articles (these may often be provide  d by review commissioners or subject  experts or have been selected for the trial critical appraisal’ | Pg 30  ‘Limiting the search by date may be used where the focus of the review is on a more recent intervention or innovation. However, potentially relevant studies as well as seminal, early studies  in the field may be missed if the limit set is too recent thus date limits should be used in an  informed way, based on knowledge of key papers relevant to the review question’  p30  ‘If limiting by language is required, it is preferable to search inclusively,  and keep a record of numbers of studies per language group. This allows the reader to identify  how many studies have been identified, but are not included, therefore promoting transparency  in the process’ | 7.1.7  ‘The languages of publication are usually restricted to those of Western Europe. However, other foreign-language publications may also be included if the available information on these publications indicates that additional and relevant information for answering the research question is to be expected.’ | Pg 81  ‘For an intervention, this involves listing the different ways in which it can be defined, perhaps by drawing up a list of synonyms. Then the population is specified, and the outcome of interest – again, including relevant synonyms.’  ‘For social systematic reviews of effectiveness the range of eligible study designs may be wide. Searching for controlled trials alone may either uncover few studies or may not identify other relevant evaluative research, and is likely to exclude studies reporting on process and implementation issues. Similarly, evaluations of the effects of social policies may involve randomized and non- randomized controlled studies, but a range of other study designs and search terms will be relevant depending on the study question.’ | 2.3.3  PICOs  ‘The research question is commonly broken into concepts, and only the most important ones are used to develop the search strategy’  ‘ For more complex review questions, it may be necessary to use several combinations of search concepts to capture a review topic’  ‘If search strategies are limited, for example, by language or publication year, this should be justified in the methods section of the systematic review. However, such limits should be used with caution, as they may introduce bias [3,4,10]. Moreover, they should only be considered if they can be reliably applied’ | Pg 23  ‘The structure of a search strategy should be based on the main concepts being examined in a review.’  P24  ‘Generally speaking, a search strategy to identify intervention studies will typically have three sets of terms: 1) the condition of interest, i.e., the population; 2) the intervention(s) evaluated; and 3) the outcomes (optional). Limiting commands may be used to further narrow the results by study design (or document type), dates, language, etc’. | 5.4  ‘Review questions can be broken down into different concepts, which can be combined to devise a search strategy. For example, the PICO (population, intervention, comparator and outcome) or the SPICE (setting, perspective, intervention, comparison, evaluation; Booth 2004) framework can be used to structure a search strategy.’  ‘When the relevant literature for a question is less well defined or indexed, a multi‑stranded approach to searching may be more efficient. This involves developing several shorter search strategies (strands) with an emphasis on precision.’ |
| Database searching | Pg 17  ‘The selection of electronic databases to search will depend upon the review topic.’  ‘Due to the diversity of questions addressed by systematic reviews, there can be no agreed standard for what constitutes an acceptable search in terms of the number of databases searched. For example, if the review is on a cross-cutting public health topic such as housing and health it is advisable to search a wider range of databases than if the review is of a pharmaceutical intervention for a known health condition’ | 6.2.1.1  ‘Searches of health-related bibliographic databases are generally the easiest and least time-consuming way to identify an initial set of relevant reports of studies.’  ‘A key advantage of these databases is that they can be searched electronically both for words in the title or abstract and by using the standardized indexing terms, or controlled vocabulary, assigned to each record.’  Further guidance is provided on national and regional databases, subject-specific databases, citation indexes and grey literature databases. | Pg 36  ‘Different databases and catalogues sample different subsets of the literature, and so multiple sources should be accessed to ensure the search is comprehensive and unbiased, but avoids unnecessary duplication.’  P37  ‘Different Review Teams often have access to different resources, and so the list of  resources searched for each review will vary, but checking bibliographies and contact with  authors should help to test if relevant articles are retrieved.’ | Pg 29  ‘An initial limited search of MEDLINE and CINAHL will be undertaken followed by analysis of the text words contained in the title and abstract, and of the index terms used to describe article. A second search using all identified keywords and index terms will then be undertaken across all included databases.’ | 7.1.2  ‘The selection of databases for each product is generally based on the focus (i.e. regarding content, methods, and region) of the bibliographic databases. At least 2 large biomedical databases (e.g. MEDLINE and EMBASE) are always selected. For the preparation of health information a search for qualitative studies is additionally conducted in CINAHL and PsycInfo.’ | Pg 101  ‘The number of databases or other sources that one needs to search varies from topic to topic, and depends on the time and resources available. It also clearly depends on one’s tolerance to the risk of missing a relevant study or studies, and one’s assessment of the cost of missing it.’ | 2.3.4  ‘The production of a systematic review requires a systematic search in several bibliographic databases. For example, previous research has shown that searching MEDLINE alone is insufficient to identify all published relevant studies on the topic of interest and may produce biased results [64-67]. This is due to the fact that journal inclusion rates differ between databases [68,69]. Furthermore, the time and quality of indexing differs [65,69-71], meaning that a reference might be more difficult to find or be found with delay in some databases, but not in others.’  ‘insufficient empirical evidence is available so far on how many and which databases should be regularly searched.’ | 3.1. pg 11  ‘Social science-related subject databases are generally the best way to identify an initial set of relevant reports of studies within a specific field.’  ‘ Decisions related to which subject-specific databases are to be searched, in addition to the main field-related database, will be influenced by the topic of the review, access to specific databases, and budget considerations.’ | 5.3  ‘The selection of sources will vary according to the requirements of the review question. For reviews of the effectiveness of pharmacological interventions, the Cochrane Central Register of Controlled Trials (CENTRAL), EMBASE and MEDLINE should be prioritised for searching. For other questions, it might be as or more important to search other sources.’ |
| Supplementary searching | P17  ‘In addition to searching electronic databases, published and unpublished research may  also be obtained by using one or more of the following methods.’ | 6.2.4  ‘Conference abstracts and other grey literature can be an important source of studies for inclusion in reviews.’  ‘Efforts should be made to identify unpublished studies.’ | General guidance on methods reported in table 2 is reported. No specific guidance on aims or purpose. | No specific guidance on aims or purpose. | 7.1.6  ‘Besides bibliographical database searches, it can be useful (depending on the research question) to conduct a handsearch in selected scientific journals and proceedings of abstracts from scientific meetings. This is decided on a case-by-case basis.’ | 4.2  ‘databases are not the only source of literature, and sometimes they are not even the most useful. In many research areas, particularly in the social sciences, the bulk of the relevant evidence may not appear in journals, but will be located in reports in the ‘‘gray literature,’’ much of which may not be indexed in electronic databases. There is thus a real risk that electronic searches alone will fail to locate a good deal of relevant information.’ | General guidance on methods reported in table 2 is reported. No specific guidance on aims or purpose. | General guidance on methods reported in table 2 is reported. No specific guidance on aims or purpose. | General guidance on methods reported in table 2 is reported. No specific guidance on aims or purpose. |
| Managing references | 1.3.1.8  advocates the use of bibliographic management software | 6.5  No guidance reported | 4.1.6  guidance on bibliographic software, on downloading data where a direct export option in not available, and saving the results | No guidance reported | 4.16  guidance on bibliographic software is given | No guidance reported | No guidance reported | 6.1  guidance on bibliographic software. | 5.8  guidance on bibliographic software. |
| Documenting the search | 1.3.1.10  No specific guidance was recommend.  The following criteria were documented for repotting:  The write up of the search should include information about the databases and interfaces searched  (including the dates covered), full detailed search strategies (including any justifications for date or language restrictions) and the number of records retrieved. | 6.6  PRISMA guidance is necessary.  ‘the full search strategies for each database will need to be included in an Appendix of the review. The search strategies will need to be copied and pasted exactly as run and included in full, together with the search set numbers and the number of records retrieved. The search strategies should not be re-typed as this can introduce errors.’  ‘save locally or file print copies of any information found on the internet, such as information about ongoing trials.’ | 4.1.5  no specific reporting guidelines mentioned but a review by Mant et al was referenced.  The following criteria was documented for reporting:  Specifically, for each source  searched a record should be made of: the dates of individual searches; the full list of  search terms employed and how these were combined;  any changes to the default search  settings of the source used; the nature of the search (e.g. keywords, topics, or full texts)  and other search options (e.g. lemmatization); the  removal of duplicates if automatically  carried out when downloading results; and all the results returned by each search. | No guidance reported | No guidance reported | 7.1.8  no specific guidance was recommend.  The following criteria were documented:  All steps in the search in bibliographic databases are documented. This especially includes:  the search strategy for the databases selected  the search date  the user interface  the number of hits  after perusal of all hits: documentation of the publications judged relevant to the research question posed (citations)  after perusal of the full texts: documentation of the citations not judged relevant; alternatively, documentation of the topic-related publications that were, however, irrelevant for the report (in each case providing a reason for exclusion)  All other steps in the information retrieval procedure are also documented (e.g. correspondence with authors, queries to manufacturers). | No guidance reported | 8.1.2  no specific guidance was recommend  The following criteria were documented for reporting:  Reporting the search process in the review:  List all databases searched;  Note the dates of the last search for each database AND the period searched;  Note any language or publication status restrictions;  List grey literature sources;  List individuals or organizations contacted;  List any journals and conference proceedings specifically handsearched for the review;  List any other sources searched (e.g. reference lists, the internet). | 5.9  specific criteria were documented for reporting.  the following information should be  documented:  date(s) on which the searches were carried out, including the date(s) of any re-run  searches (see  section 5.10)  names of the databases, database host systems and database coverage dates  names of any other sources searched  search strategies for all sources,  annotated to explain any  decisions on included  and excluded terms which are not self  -explanatory  details of any supplementary searching undertaken,  including  the rationale  any limits or search filters applied to the search  (for example,  language, date, study design). |

Key: * These handbooks focus on specific guidance (as per their topic) in the first instance. Subsequent chapters within the handbooks focus on other review topics. These include: reviews of diagnostic and prognostic studies, review of public health topics and reviews of qualitative studies. These chapters develop the primary guidance accounting for peculiarities specific to the topic. I have not focused on these additional topics here.

**PubMed search strategy**

Database: PubMed

Host: National library for medicine (NLM) via: <https://www.ncbi.nlm.nih.gov/pubmed/>

Date searched: August 30^th^ 2017

Searcher: Chris Cooper

Checked by: Jo Varley-Campbell

Search strategy: (literature search*[Title/Abstract]) AND sysrev_methods[sb]

Results: 586
